# Multifocal Posterior Pigment Epitheliopathy Complicated by Circumferential Choroidal Detachment Treated With Fluorescein Angiography-Guided Focal Laser Photocoagulation: A Case Report

**DOI:** 10.7759/cureus.105139

**Published:** 2026-03-12

**Authors:** Mai Honda, Akira Watanabe, Daisuke Shinohara, Kokoro Konuma, Tadashi Nakano

**Affiliations:** 1 Ophthalmology, The Jikei University School of Medicine, Tokyo, JPN

**Keywords:** choroidal detachment, multifocal posterior pigment epitheliopathy, retinal photocoagulation, retinal pigment epithelium, serous retinal detachment

## Abstract

Multifocal posterior pigment epitheliopathy (MPPE) is a pachychoroid-related disorder characterized by choroidal hyperpermeability and serous retinal detachment (SRD), typically without choroidal detachment (CD). We report a rare case of MPPE complicated by circumferential CD in a man in his late 50s with chronic myeloid leukemia in molecular remission. Fundus examination revealed multiple yellowish-white exudative lesions with SRD at the posterior pole and peripheral CD. Fluorescein angiography (FA) demonstrated multiple focal leakage points with progressive dye leakage, while indocyanine green angiography showed early hypofluorescence followed by late hyperfluorescence. Although the presence of CD suggested possible overlap with uveal effusion, the overall clinical and angiographic findings were predominantly consistent with MPPE. Focal laser photocoagulation was applied to angiographically identified leakage sites; however, repeat angiography revealed newly developed leakage points requiring additional treatment. Both SRD and CD gradually resolved, with complete resolution five months after therapy. This case suggests that localized MPPE-related leakage may have played a predominant role in the development of circumferential CD and highlights the importance of repeated angiographic evaluation and FA-guided treatment in complex or atypical presentations of pachychoroid-related disorders.

## Introduction

Multifocal posterior pigment epitheliopathy (MPPE), originally described in Japan by Uyama, is characterized by marked choroidal hyperpermeability leading to disruption of the outer blood-retinal barrier at the level of the retinal pigment epithelium (RPE) and subsequent accumulation of subretinal exudative fluid [1]. Clinically, MPPE typically presents with multiple exudative lesions approximately one disc diameter in size located in the posterior pole, usually without associated choroidal detachment (CD) [1,2]. Fluorescein angiography (FA) demonstrates intense focal leakage corresponding to these lesions [1-3].

In the Western literature, a similar clinical presentation has been described as bullous retinal detachment and is generally regarded as a severe manifestation of central serous chorioretinopathy (CSC) [4]. MPPE predominantly affects middle-aged men, frequently presents bilaterally, and is often associated with known risk factors for CSC, including a history of CSC, renal disease, and systemic corticosteroid use [1-3,5,6]. With advances in multimodal imaging, MPPE is increasingly considered part of the pachychoroid disease spectrum, which encompasses CSC and related disorders characterized by increased choroidal thickness, dilated outer choroidal vessels, and choroidal hyperpermeability [7].

Within the pachychoroid spectrum, MPPE shares several clinical and imaging features with CSC, including serous retinal detachment (SRD) and focal leakage on FA. However, MPPE is typically characterized by multiple posterior pole exudative lesions approximately one disc diameter in size and more extensive subretinal fluid accumulation, whereas CSC more commonly presents with localized SRD and a limited number of leakage points.

Idiopathic uveal effusion (UE) syndrome, first described by Schepens et al. in 1963, is characterized by a highly mobile, non-rhegmatogenous (exudative) retinal detachment accompanied by circumferential ciliochoroidal detachment [8]. UE is associated with various underlying conditions, including abnormalities of scleral permeability, nanophthalmos, ocular hypotony following trauma or surgery, inflammatory disorders, primary angle-closure glaucoma, intraocular malignancy, and disturbances in choroidal circulation [9]. Based on the presence or absence of nanophthalmos and scleral thickening, UE syndrome has been classified into three clinical types [9].

Although uncommon, MPPE may occasionally be complicated by CD, and such cases have been suggested to show overlapping features with UE [3]. Because both MPPE and UE involve abnormalities in choroidal circulation and fluid dynamics, overlapping clinical manifestations may occur. However, MPPE complicated by circumferential CD is extremely rare, and its clinical characteristics and optimal management have not been well described in the literature.

Here, we report a case of MPPE complicated by circumferential CD in which FA-guided focal laser photocoagulation resulted in clinical improvement.

## Case presentation

A man in his late 50s with a history of chronic myeloid leukemia (CML) presented to a local ophthalmology clinic with blurred vision in his right eye. Fundus examination revealed circumferential CD in the peripheral retina and SRD in the posterior pole of the right eye. The patient was initially diagnosed with Vogt-Koyanagi-Harada (VKH) disease at another institution and was treated with intravenous methylprednisolone pulse therapy (1,000 mg/day for three consecutive days), followed by oral prednisolone at 60 mg/day for two days; however, no clinical improvement was observed.

**Figure 1:**
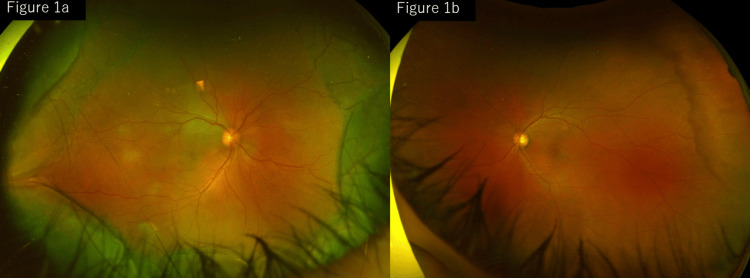
Fundus photographs at presentation: (a) right eye and (b) left eye. At presentation, the right eye shows multiple yellowish-white exudative lesions at the posterior pole with SRD and circumferential CD in the peripheral fundus (a). The left eye shows subtle parafoveal color changes without a definite SRD (b). CD, choroidal detachment; SRD, serous retinal detachment

At the initial visit to our hospital, the best-corrected visual acuity was 0.5 in the right eye and 0.9 in the left eye. The intraocular pressure was 17 mmHg in both eyes, as measured using non-contact (air-puff) tonometry. A shallow anterior chamber was observed in the right eye; however, no anterior chamber inflammation was observed in the other eye. Fundus examination of the right eye revealed multiple ill-defined, yellowish-white exudative lesions, predominantly at the posterior pole, accompanied by SRD. Circumferential CD was also observed in the peripheral fundus. Subtle color changes were noted in the parafoveal region of the left eye, although no definite SRD was detected (Figure 1).

**Figure 2:**
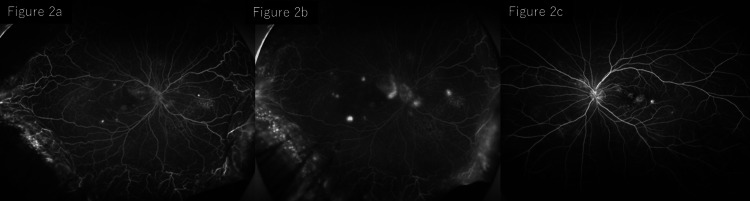
FA at presentation: (a) early phase and (b) late phase of the right eye; (c) late phase of the left eye. FA showing multiple pinpoint leakage sites at the posterior pole of the right eye in the early phase, (a) with progressive leakage in the late phase (b). In the left eye, several pinpoint leakage sites are detected near the macula (c). FA, fluorescein angiography

FA revealed multiple pinpoint hyperfluorescent spots corresponding to exudative lesions at the posterior pole of the right eye during the early phase. These leakage points gradually increased over time, indicating progressive dye leakage. Several pinpoint leakage sites were detected near the macula in the left eye (Figure 2).

**Figure 3:**
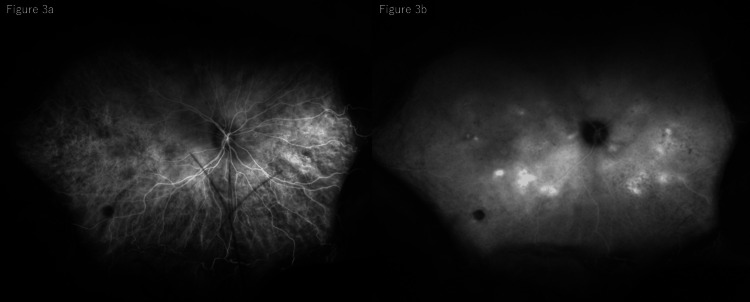
Indocyanine green angiography at presentation: (a) early phase and (b) late phase. Indocyanine green angiography demonstrating early hypofluorescent spots corresponding to leakage sites, (a) followed by late hyperfluorescence (b). Evaluation of choroidal vessels is limited because of extensive CD. CD, choroidal detachment

Indocyanine green angiography (ICGA) showed early hypofluorescence corresponding to the leakage sites observed on FA, followed by late hyperfluorescence involving the surrounding areas. Choroidal vascular dilation was difficult to evaluate due to extensive CD (Figure 3).

**Figure 4:**
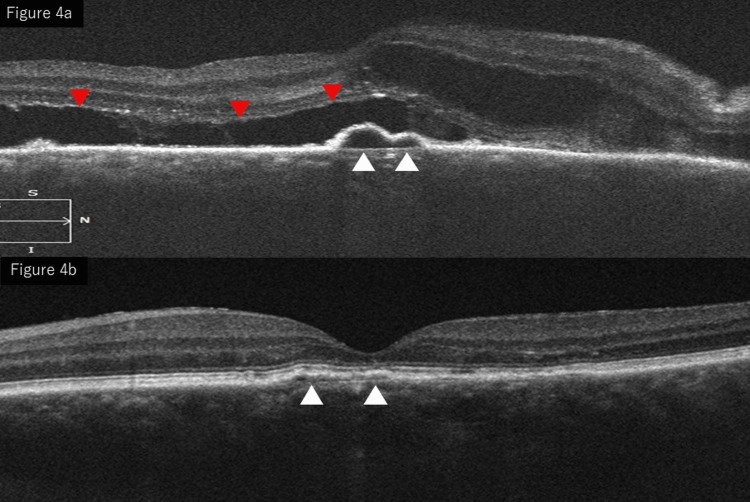
OCT at presentation. OCT of the right eye shows multiple PEDs (white arrows), SRD, macular edema, and choroidal thickening (red arrows) (a). The left eye shows RPE irregularities and outer retinal disruption (white arrows) (b). SRD, serous retinal detachment; RPE, retinal pigment epithelium; OCT, optical coherence tomography; PEDs, pigment epithelial detachments

Optical coherence tomography (OCT) of the right eye revealed multiple pigment epithelial detachments (PEDs), SRD, macular edema (ME), and marked choroidal thickening. Irregularities in the RPE and disruption of the outer retinal layers were observed in the left eye. The central retinal thickness within the central 1 mm area measured 633 μm in the right eye and 218 μm in the left eye. Enhanced depth imaging optical coherence tomography (EDI-OCT) was used to assess the choroid. The subfoveal choroidal thickness measured 430 μm in the right eye and 425 μm in the left eye, indicating bilateral choroidal thickening. However, dilated pachyvessels were not clearly identifiable on the available images (Figure 4).

The axial length of both eyes was 24 mm, and B-scan ultrasonography revealed no evidence of scleral thickening. The patient’s CML had been in remission for five months following treatment with nilotinib at the time of presentation, and an association with the ocular findings was considered unlikely. Molecular monitoring using real-time quantitative polymerase chain reaction (RQ-PCR) demonstrated a BCR-ABL1 (International Scale) value of ≤0.1%, indicating maintenance of a major molecular response, and hematological parameters remained within normal limits.

Although CD is atypical, the condition was considered to be predominantly MPPE. Therefore, focal retinal photocoagulation was applied to the leakage sites identified on FA using a multicolor laser (yellow; spot size 100 μm, duration 100 ms, power 130 mW, approximately eight shots per leakage point). However, SRD worsened thereafter. Considering the possible contribution of nilotinib, the medication was discontinued; however, no improvement was observed during the three months following laser treatment.

**Figure 5:**
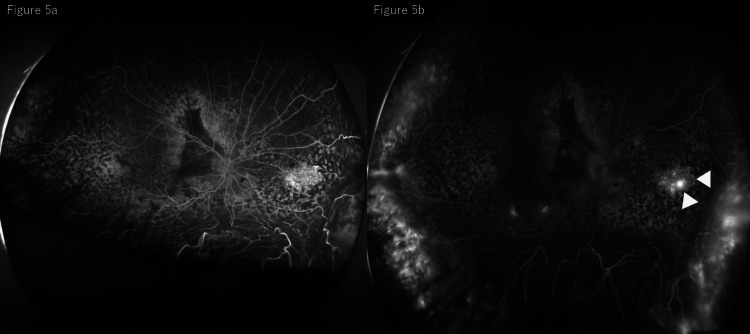
FA after initial photocoagulation: (a) early phase and (b) late phase. After focal retinal photocoagulation, FA shows resolution of the leakage at the treated sites with new leakage points on the nasal side (white arrow) and leopard-spot-like hyperfluorescence in the late phase. FA, fluorescein angiography

Repeat FA performed three months after the initial laser treatment showed resolution of the leakage at the previously photocoagulated sites, while new leakage points were identified on the nasal side. In addition, leopard-spot-like hyperfluorescence was observed in the late phase (Figure 5).

**Figure 6:**
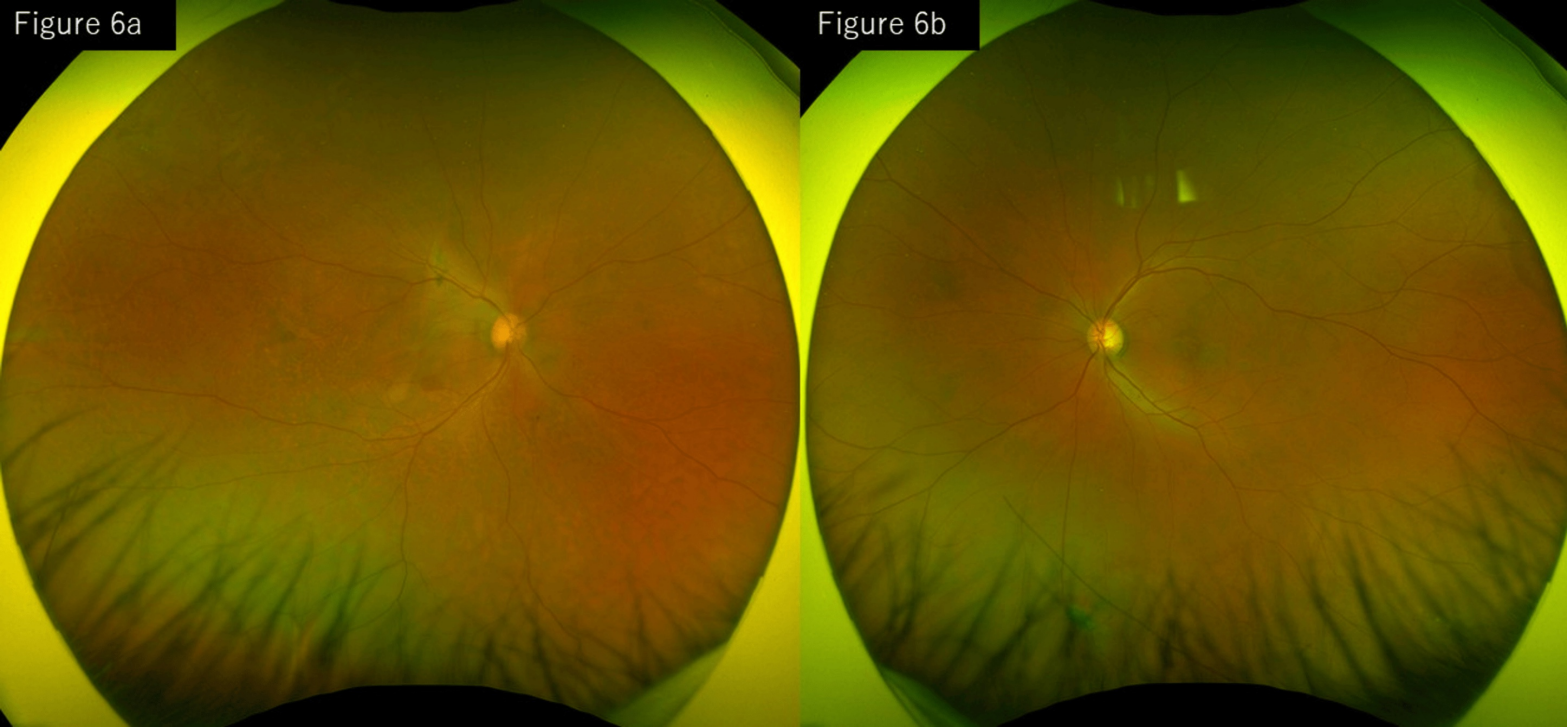
Follow-up images after treatment: (a) right eye and (b) left eye. Five months after additional photocoagulation, fundus photographs and OCT show complete resolution of the SRD and CD. CD, choroidal detachment; SRD, serous retinal detachment; OCT, optical coherence tomography

Additional retinal photocoagulation was applied to the remaining leakage sites using a multicolor laser (yellow; spot size 100 μm, duration 100 ms, power 130 mW, nine shots). Three months later, both the SRD and CD showed a tendency toward resolution, and complete resolution was achieved five months after treatment. The central retinal thickness within the central 1 mm area improved to 283 μm in the right eye and 216 μm in the left eye (Figure 6).

## Discussion

The patient presented with clinical features characteristic of MPPE and UE. Findings typical of MPPE include multiple yellowish-white exudative lesions in the posterior pole with associated SRD, as well as multiple focal leakage points with surrounding subretinal fluid accumulation on FA. In addition, features consistent with UE were observed, including SRD, circumferential peripheral ciliochoroidal detachment, and a leopard-spot pattern on FA. These findings suggest an overlap between MPPE and UE; however, the precise pathophysiology in this case remains unclear [3].

The patient was initially diagnosed with VKH disease at another institution and was treated with steroid pulse therapy without clinical improvement. Previous reports have described the development of CSC-related disorders after corticosteroid therapy in patients with UE [5]. In the present case, although steroid pulse therapy did not improve the condition, neither the SRD nor the fluorescein leakage worsened during treatment. Therefore, a direct contribution of corticosteroids to the disease process is unlikely. Nevertheless, careful differentiation from CSC-related disorders is essential when considering steroid therapy. The absence of intraocular inflammation, the lack of typical VKH-related ICGA findings, and the poor response to corticosteroid therapy render VKH disease unlikely.

The patient had been receiving nilotinib therapy for CML for three years and six months prior to presentation, prompting consideration of whether either CML itself or nilotinib therapy could have contributed to the ocular findings. Although tyrosine kinase inhibitors have been reported to cause several ocular adverse effects, including periorbital edema and retinal vascular events, a direct association between nilotinib and pachychoroid-related disorders has not been established. In this case, discontinuation of nilotinib did not lead to improvement of the ocular findings, and the drug was successfully resumed after laser photocoagulation without recurrence. Therefore, although a potential contribution of systemic disease or medication cannot be entirely excluded, the clinical and angiographic findings were more consistent with pachychoroid-related pathology.

Previous studies have reported that MPPE may occasionally be accompanied by circumferential CD and have been described as occupying an intermediate position between MPPE and UE [3]. Although the mechanisms underlying CD differ between the two conditions, MPPE is primarily attributed to increased choroidal vascular permeability, whereas UE is attributed to impaired scleral permeability. Uemura proposed that MPPE represents a localized pathology extending from the RPE to the retina, whereas UE without scleral abnormalities reflects diffuse involvement of the full thickness of the choroid [9]. Furthermore, overlapping cases involving a continuous pathology from the RPE to the entire choroid have been described [10]. Koizumi et al. suggested a possible pathophysiological overlap between MPPE and idiopathic UE [7].

Given the absence of scleral thickening or nanophthalmos, focal laser photocoagulation was initially performed. Scleral window surgery has been reported to be an effective treatment for UE; however, its efficacy is limited to type three cases without nanophthalmos or scleral thickening [10]. Conversely, a 65-year-old man with bilateral pachychoroid disease and chronic CSC in the fellow eye developed posterior pole serous SRD and circumferential peripheral CD, for whom photodynamic therapy was ineffective; however, repeated scleral window surgery with full-thickness sclerectomy combined with mitomycin C resulted in improvement [10].

In contrast, several reports have demonstrated improvement of CD in MPPE complicated by UE following laser photocoagulation of retinal leakage points [1,3,11]. Currently, no consensus exists on whether laser photocoagulation or scleral window surgery is more effective for MPPE complicated by UE. In this case, pachychoroidal features were observed, and both serous retinal and CD improved following laser photocoagulation. This suggests that focal leakage from localized lesions, rather than impaired scleral permeability, played a predominant role in the development of CD. Enhanced depth imaging OCT demonstrated increased subfoveal choroidal thickness (430 μm in the right eye and 425 μm in the left eye), supporting a pachychoroid phenotype. However, distinct pachyvessels were not clearly identifiable in this case.

However, a single treatment session was insufficient, underscoring the need for repeated FA to confirm adequate treatment of existing leakage points and to detect newly developed leakage sites. If insufficient improvement is achieved, scleral window surgery may be considered as an additional option.

Both laser photocoagulation and scleral window surgery have been reported to require multiple treatment sessions to achieve clinical improvement [3,10]. Therefore, in cases refractory to the initial treatment, reassessment with FA is essential. Based on disease activity, either repeated application of the same modality or the addition of alternative surgical approaches should be considered.

Further accumulation of similar cases and multimodal imaging analyses will be necessary to better clarify the pathophysiological relationship between MPPE and UE in such atypical presentations.

## Conclusions

This case suggests a possible overlap between MPPE and UE. Resolution of circumferential CD following improvement of FA-confirmed focal leakage after repeated focal laser photocoagulation indicates that MPPE-related pathology may have played a predominant role in this case. However, overlapping mechanisms related to UE cannot be entirely excluded. The requirement for multiple laser sessions highlights the importance of careful multimodal imaging assessment and FA-guided treatment in managing atypical presentations of pachychoroid-related disorders.
